# Improvement of corn stover fuel properties via hydrothermal carbonization combined with surfactant

**DOI:** 10.1186/s13068-019-1581-x

**Published:** 2019-10-17

**Authors:** Ren Tu, Yan Sun, Yujian Wu, Xudong Fan, Jiamin Wang, Shuchao Cheng, Zhiwen Jia, Enchen Jiang, Xiwei Xu

**Affiliations:** 1 College of Materials and Energy in South China Agricultural University, Guangzhou, 510640 China; 2 State Key Laboratory of Pulp and Paper Engineering, South China University of Technology, Guangzhou, 510640 China; 3 Inst Chem & Bioengn, Swiss Fed Inst Technol, Zurich, Switzerland

**Keywords:** Combustion, Fuel properties, Hydrothermal carbonization, Kinetic analysis, Pelletization, Solid product, Surfactant

## Abstract

**Background:**

Biomass fuel has been used to supply heat or crude materials in industry to replace the traditional fossil fuel which was one of the chief causes of climate warming. However, the large-scale utilization of biomass fuel was restricted due to the low density and high hydrophilicity of biomass, which causes the problem of transportation and storage. Therefore, pelletization of biomass was used to improve its fuel density. At present, the biomass pellet was widely used to supply heat, gas or electricity generation via gasification, which supplied clean and sustainable energy for industry. However, the energy consumption during pelletization and high hydrophilicity of pellets were still the problem for the large-scale application of biomass pellet. In this study, hydrothermal carbonization and surfactant played the role of permeation, adsorption and wetting in the solution, which was expected to improve the fuel properties and pelletization effectivity of corn stover.

**Results:**

In the article, surfactant (PEG400, Span80, SDBS) was chosen to be combined with wet torrefaction to overcome the drawbacks and improve the pelletization and combustion properties of Corn stover (CS). Especially, hydrothermal carbonization (HTC) combined with surfactant improves the yield of solid products and reduces the ash content of solid product, which was beneficial for reducing the ashes of furnace during gasification. Meanwhile, surfactant promotes the formation of pseudo-lignin and the absorption for oil with low O and high C during HTC, which improves the energy density of solid product. Furthermore, the oil in solid product plays the role of lubricant and binder, which reduces the negative effect of high energy consumption, low bulk density and weak pellets strength caused by HTC during pelletization. HTC combined with surfactant improved the hydrophobicity of pellet as well as grindability due to the modification of solid product. Moreover, surfactant combined with HTC improved the combustion characteristic of solid product such as ignition and burning temperature as well as kinetic parameters due to the bio-oil absorbed and the improvement of surface and porosity.

**Conclusions:**

The study supplied a new, less-energy intensive and effective method to improve the pelletization and combustion properties of corn stover via hydrothermal carbonization combined with surfactant, and provided a promising alternative fuel from corn stover 
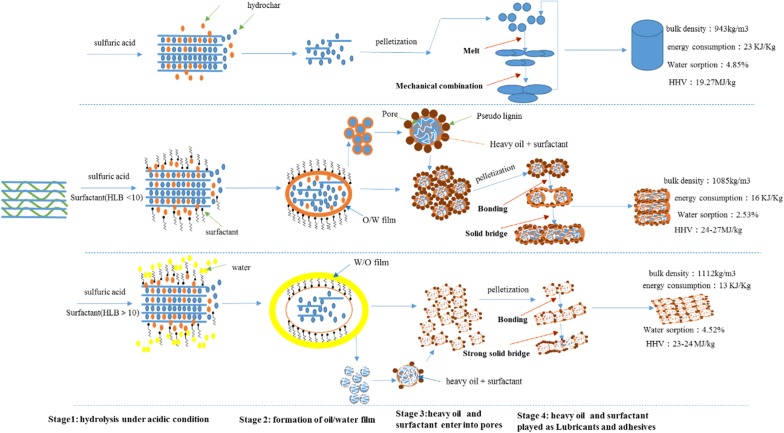
.

## Highlights

1. HTC combined with surfactant to improve the pelleting and combustion behavior of CS.
2. Surfactant promotes formation of pseudo-lignin and absorption for oil with low O and high C.
3. Oil on hydrochar improved bulk density, energy density and decreased energy consumption.
4. The combustion properties of CS/SA/PEG is similar with diesel oil.
5. Mechanism of pelletization and combustion improvement of CS via HTC with surfactant.

## Background

Nowadays, the renewable and sustainable energy was widely studied due to the environmental problems from fossil fuel. Corn stover (CS) was a common agriculture waste, whose yield was 1.8 * 10^7^ t in 2017 in China. Part of CS was used in the world as a renewable materials for producing chemicals and fuel due to the low price and wide availability [1, 2]. However, the utilization ratio was low in China. A large amount of corn stover was direct combustion, resulting in waste of resources and environmental pollution. It was promising to realize the high value utilization of CS in China as renewable energy.

Moreover, with the development of biomass processing technology, biomass fuel has been used to supply heat or crude materials in industry to replace the traditional fossil fuel due to the climate warming. However, the large-scale application of biomass fuel was restricted due to the low density and high hydrophilicity of biomass, which causes the problem of transportation and storage. Therefore, pelletization of biomass was used to improve the fuel density [3, 4]. However, the high energy consumption, the high hydrophilicity and low bulk density of the pellets were also intractable problems for biomass pelletization.

Currently, all kinds of pretreatment methods such as torrefaction and hydrothermal carbonization (HTC) were introduced to combine with biomass pelletization to improve the energy density and extend the storage time [5].

HTC involving reactions under mild temperatures (120–280 °C) with self-generated pressure was widely considered as the promising method for improving the energy density and hydrophobicity [6, 7]. Therefore, combined HTC and pelleting was used to further improve the energy density, densification and hydrophobicity. However, after torrefaction, the energy consumption of biomass pelletization significantly increased due to the removal of materials with rich H– (hydrogen bond) in biomass [8]. The bulk density and radial compressive strength of biomass pellets also significantly decreased after HTC [8, 9]. Those significantly decreased the effectivity and improved the expense of biomass pelletization. Moreover, the combustion properties such as ignition temperature and the burnout temperature shifted to the high temperature zone after HTC [10]. Those were also the key points for restricting the development of high-quality pellets fuel in the industrial application. Furthermore, the pelletization mechanism of wet-torrefied pellets was still not clear. Surfactant had fixed hydrophilic and lipophilic groups, which can significantly decrease the surface tension of the solution. In our previous study, surfactant combined with HTC significantly improved the characteristics of solid product by promoting the polycondensation of pseudo-lignin and the deposition of heavy oil in solid product [11].

In this article, five different surfactants such as Tween80 (TW), Span80 (SP), sodium dodecylbenzenesulfonate (SDBS), sodium lignosulfonate (SL), PEG 400 (PEG) were added during HTC. The chemical and physical properties of solid product were analyzed. Moreover, the pelleting properties of solid product were analyzed as well as the combustion properties. Meanwhile, the mechanism of improvement of pelletization and combustion behavior of corn stover via hydrothermal carbonization combined with surfactant was also investigated.

## Result

### Physical and chemical properties of solid product obtained from corn stover

#### Distribution of hydrothermal products

**Fig. 1:**
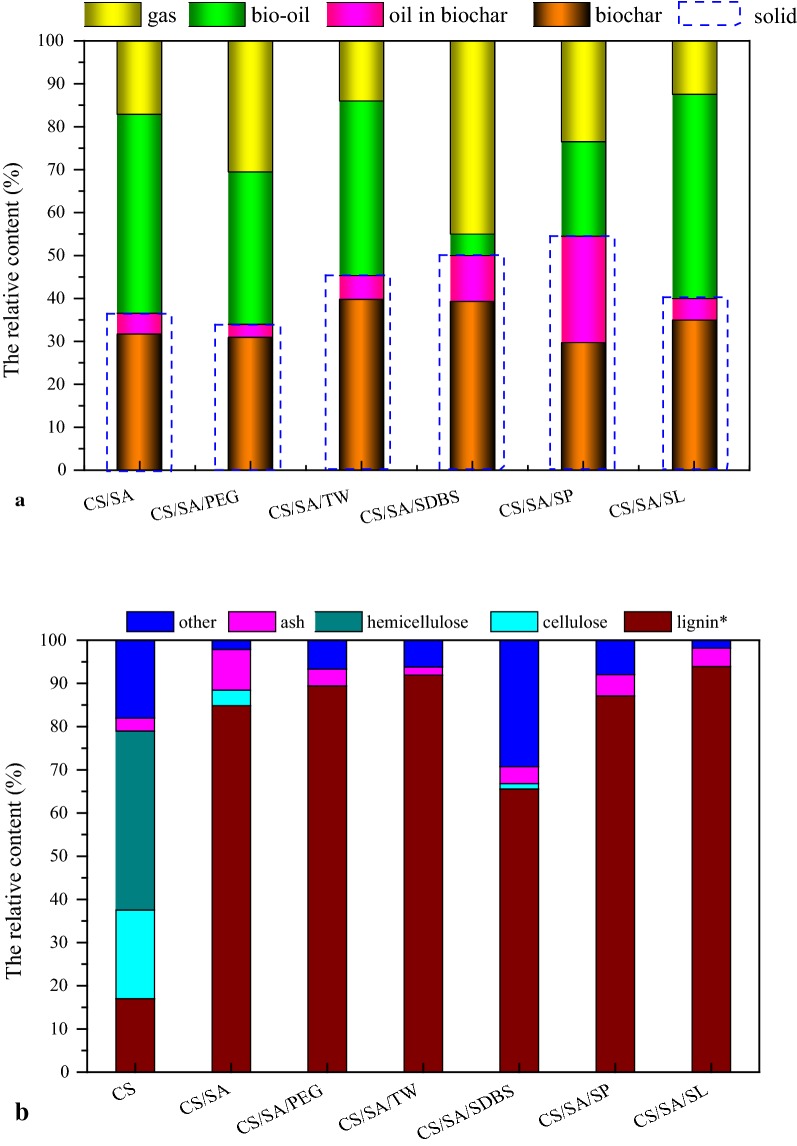
The distribution of hydrothermal products and components of solid product. **a** Distribution of hydrothermal products. **b** The components of CS and solid product obtained via HTC with different surfactant (lignin* was the lignin in the hydrochar contained the pseudo lignin and lignin)

The influence of adding different surfactant during HTC on the products distribution is shown in Fig. 1a. The yield of solid product obtained by HTC without surfactant was only 36.6%. The content of biochar and the oil in solid product was 31.73% and 4.83%, respectively. The main product was bio-oil, reaching 46.3%. However, when adding different surfactants, the products distribution was changed significantly. Especially, when adding PEG400, the solid yield slightly decreased. But the biochar content was similar to the control one (CS/SA). The bio-oil decreased from 46.3 to 35.5%, indicating that PEG400 promoted the conversion of bio-oil into gas. Moreover, when adding Tween80, the solid products content increased from 36 to 54%, indicating that the Tween80 inhibited the hydrolysis of CS. Especially, the biochar content in the solid products increased from 31.73 to 39.89%. It was possible that Tween80, acting as a hydrophilic surfactant, formed a water film on surface of the particle during HTC. The water film inhibited the releasing of oil from the particle, which restricted the decomposition of CS. Therefore, the liquid and gas products were less than the HTC of CS without surfactant. Similar, adding SDBS also inhibited the HTC of CS. The solid product content increased from 36.56 to 50.00%. The gas content increased from 17.15 to 45.00% but liquid content decreased from 46.30 to 5.00%, inducing that adding SDBS promoted the conversion of bio-oil into gas. Meanwhile, the oil in solid product significantly increased from 4.83 to 10.68%. It was also possible that the SDBS, which was a hydrophilic surfactant, formed a water film on the surface of sample, preventing the releasing of bio-oi from solid product. But the gas can easily come out from the film.

When adding sodium lignosulfonate (SL), the products distribution was similar with CS/SA, indicating that the SL had slight effect on the HTC of CS. Noticeably, the solid products were highest when adding Span80 during HTC. Especially, the oil in solid product increased from 4.83 to 24.79%. Biochar slightly decreased from 31.73 (CS/SA) to 29.71%, indicating that Span80 promoted the hydrolysis of CS. It was possible that hydrophobic surfactant Span80 gradually formed an oil film on surface of the particle during HTC. The bio-oil from the hydrolysis of biomass entered into the oil film fast, which enhances the decomposition of CS. The oil film became thicker and thicker with the hydrolysis of CS. Moreover, part of Tween80 (polyoxyethylene sorbitol fatty acid esters) cracked and formed the fatty acid and other organic materials which dissociated in the solution. When adding NaOH, it formed fatty acid sodium which was absorbed on the solid product and increased the oil in solid product.

The cellulose, hemicellulose and lignin content of CS and solid product is shown in Fig. 1b. The lignin content of CS was only 17.35%. After HTC with acid, acid and surfactant, the content of lignin in solid product was significantly improved. It was because that dilute acid promoted the hydrolysis of cellulose and hemicellulose [12]. The relative lignin content of CS/SA, CS/SA/TW, CS/SA/SP, CS/SA/PEG, CS/SA/SDBS and CS/SA/SL was 84.85%, 91.90%, 87.11%, 89.42% and 93.92%, respectively. In Fig. 1a, the solid yield of these samples was 31.73%, 39.81%, 29.71%, 30.97%, 39.32% and 34.99%, respectively. Combined Fig. 1a, b, it can be calculated that the yield of lignin was 267.8 mg/g CS, 365.9 mg/g CS, 258.8 mg/g CS, 276.9 mg/g CS, 257.8 mg/g CS, 328.7 mg/g CS, respectively. However, the content of lignin in CS was only 160 mg/g CS. Those data induced that the pseudo-lignin was produced during HTC and surfactant Tween80 and SDBS promoted the formation of pseudo-lignin. Moreover, Demirbas et al. [13] found that the higher the lignin content was, the higher the heat value (HHV) was in materials.

Especially for CS/SA/TW, the lignin yield was twice as high as CS. Compared with CS/SA, Tween80 and SL significantly improved the yield of pseudo-lignin. Sannigrahi et al. [14] also found that the formation of pseudo-lignin was obtained from the combination of carbohydrate. Lignin degradation products were responsible for the increase of Klason lignin content in biomass under acidic conditions.

#### The fuel properties of corn stover solid product

**Fig. 2:**
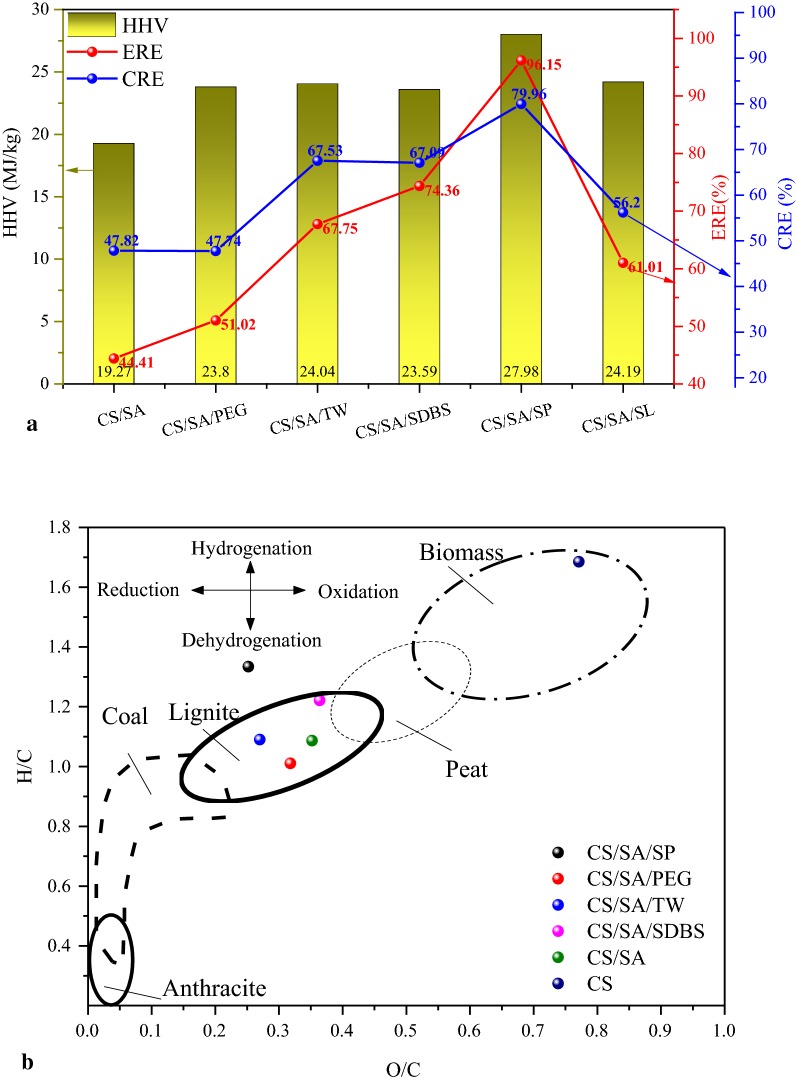
The fuel properties of solid product and CS. **a** The HHV of solid product, ERE and CRE during HTC. *CRE* carbon recovery efficiency (%), *ERE* energetic retention efficiency (%). The calculation formula of energy parameter is shown in Table 5. **b** Van Krevelen diagram for CS and torrefied solid product

It can be seen from the Table 1 and Fig. 2a that the carbon recovery efficiency (CRE) and solid recovery (SR) were in the order: CS/SA/PEG < CS/SA < CS/SA/SL < CS/SA/TW < CS/SA/SDBS < CS/SA/SP. Especially, the CRE was 67.53%, 67.09% and 79.96% for CS/SA/TW, CS/SA/SDBS and CS/SA/SP, respectively. Noticeably, the CRE increased from 47.82 to 79.96% after adding Span80 during HTC. However, fixed carbon recovery efficiency was 87.27%, which was similar to the sample of CS/SA. It was that adding Span80 promoted the adsorption of bio-oil with low oxygen and high carbon, which improved the CRE. Moreover, after adding PEG during HTC, the CRE and fixed carbon recovery efficiency were similar to CS/SA sample and the oil in char was also similar to CS/SA. However, the HHV improvement significantly increased. It was possible that PEG promoted the deoxidization and dehydrogenation during HTC and improves the HHV. The conclusion was consistent with the results in Fig. 2 (Van krevelen). Similar, Tween80 and SL promoted the deoxidization during HTC, resulting in the increase of HHV.

**Table 1:** Derived parameter obtained from solid yield, elemental analysis, proximate analysis and heat value of hydrothermal solid product

Sample	Solid recovery (%)	Carbon recovery efficiency (%)	Fixed carbon recovery efficiency (%)	Energy densification efficiency (%)	Energetic retention efficiency (%)	HHV improvement (%)
CS/SA	36.6	47.82	88.59	121.50	44.41	21.50
CS/SA/PEG	34.0	47.74	83.87	150.06	51.02	50.06
CS/SA/TW	44.7	67.53	100.33	151.58	67.75	51.58
CS/SA/SDBS	50.0	67.09	113.77	148.74	74.36	48.74
CS/SA/SP	54.5	79.96	87.27	176.42	96.15	76.42
CS/SA/SL	40.0	56.20	100.28	152.52	61.01	52.52

Moreover, the energy densification efficiency, energetic retention efficiency and HHV improvement were significantly improved after adding surfactant. Especially, the increase ratio for CS/SA/SP sample was 55%, 52% and 55%, respectively. Combined with the results in Fig. 1a, it can be seen that the oil in the solid product reached 45%, which was much higher than the CS/SA sample. It was possible that Span80 formed an oil film on surface of the solid product during HTC, which significantly enhanced the absorption for bio-oil with lower O and high C, resulting in the improvement of energy densification efficiency and HHV. On the other side, the addition of surfactant Span80 during HTC may promote both the dehydration and decarboxylation resulting in the increase of HHV.

Figure 2b showed the Van Krevelen diagram which was used to show the elemental composition, the possible reaction pathways for CS hydrolysis and the fuel quality of solid product [15]. It can be seen that both of H/C and O/C ratio significantly decreased after HTC combined with different surfactant, indicating that CS was converted into carbonaceous products via reduction and dehydration during HTC [16, 17]. It was widely accepted that the products with higher energy would be placed at the lower left side and closed to the origin of Van Krevelen diagram. All of solid product from HTC combined with the PEG, Tween80 and SDBS were in the range of lignite, excepting for solid product with adding Span80 [18]. It was possible that the oil in the solid product of Span80 was much higher than the other solid product and the composition of oil was with high H content. Those indicated that the fuel value of CS was enhanced after HTC. Moreover, it was well known that the hydrogen and oxygen content would be reduced via dehydration and decarboxylation. It was obvious that when adding PEG, TW or SP, the O content in solid product was lower than it in solid product without surfactant. It indicated that surfactant promoted the decarboxylation of CS during HTC.

The proximate and heat value analysis of solid product samples are shown in the Table 2. It was obvious that, compared with CS raw materials, the volatile matter content decreased significantly due to the decomposition of hemicellulose and cellulose during HTC. Especially, the volatile matter content was lower in CS/SA, CS/SA/PEG and CS/SA/SL. It was because that all hemicellulose and most of cellulose were removed from the CS after HTC and the oil absorbed on the solid product was low in the solid product. The results were consistent with the content of oil in solid product in Fig. 1a, inducing that the oil in char was also contributed to the volatile of samples. The ash, which will cause slagging, was one of the key factors for elevating the combustion properties of solid product. Compared with CS/SA, after adding surfactant during HTC, the ash content decreased significantly from 7.23% to about 3% for most of solid product. It was possible that surfactant was beneficial for the removal of ash in samples. For CS/SA, the ash content was higher than CS. This means that the decomposition amount of organic matter in CS shell during HTC was high than the mass loss of metal in CS. Moreover, fixed carbon was the key factor for contributing heat. The content of fixed carbon was high in the sample of CS/SA/PEG (40.58%) and CS/SA/SL (41.24%), indicating that PEG and SL were favorable for the formation of fixed carbon and improvement of heat value. It was accepted that the higher the fixed carbon was, the higher the heat value was. Noticeably, the fixed carbon of CS/SA/SP was not high, but the heat value reached the highest (27.98 MJ/kg) in the solid products. It was possible that the oil in the solid product with high C and low O, which accounts for 45.49% of the solid product, improved the heat value.

**Table 2:** Proximate analysis of solid product and CS

Sample	Moisture content (%)	Ash content (%)	Volatile matter (%)	Fixed carbon (%)
CS	8.01	1.71	73.83	16.45
CS/SA	4.44	6.19	49.51	39.86
CS/SA/TW	2.85	6.93	53.81	36.41
CS/SA/SP	3.80	3.70	66.16	26.34
CS/SA/PEG	3.80	3.79	51.83	40.58
CS/SA/SDBS	5.44	3.52	53.61	37.43
CS/SA/SL	3.12	4.04	51.60	41.24

#### The FT-IR analysis of solid product obtained from HTC associate with surfactant

The FT-IR figure is in Additional file 1. The infrared absorption spectrum baseline was processed according to the method of Faix [19]. FT-IR showed that all samples owned typical lignin spectral characteristics with OH–, C–H, and C=O stretching at 1615.6 cm^−1^, 1713.7 cm^−1^, 2849.9 cm^−1^, 2923.4 cm^−1^, and 3428.2 cm^−1^. The vibrations at 1505.7 cm^−1^ were corresponding to the aromatic ring skeleton. All of those induced that there was lignin structure in the solid product, which was consistent with the results of composition analysis in Fig. 1b. The band at 1109.5 cm^−1^ was assigned to the aromatic C–H deformation of S-type lignin, appearing at CS/SA/TW and CS/SA/SDBS. It induced that adding TW and SDBS inhibited the hydrolysis of lignin or promoted the formation of S-type lignin. The results were consistent with the analysis of products distribution of HTC in Fig. 1a, b. The band at 2923.4 cm^−1^ of corn stalk raw material corresponded to the C–H stretching of CH_3_ or CH_2_ in alcohol and phenolic group [20]. After the addition of surfactant, shoulder peaks appeared at the same place, which was mainly caused by asymmetrical aromatic ring C–H Symmetry. It induced that surfactant changed the symmetry of the aromatic ring.

#### The composition of oil in solid product

**Fig. 3:**
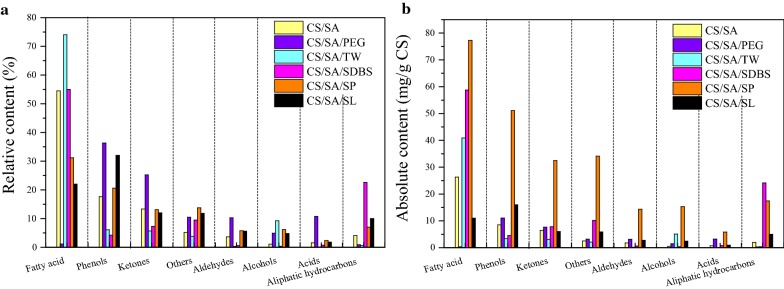
The composition of oil extracted from solid product. **a** The relative content of components in bio-oil extracted from solid product. **b** The absolute content of components in bio-oil extracted from solid product

The composition and distribution of the oil in solid product were shown in the Fig. 3. There were a small amount of ketone, aldehydes, alcohols, acids, and aliphatic hydrocarbons and so on. The composition and content of oil in solid product varied with the types of surfactant. It was possible that the bio-oil from CS hydrolysis formed W/O or O/W under the role of surfactant and caused the change of hydrolysis pathway of CS due to the difference of HLB value of different surfactant. Moreover, the adsorption of solid product for the bio-oil depends on the structure of solid product and the type of surfactant.

When adding the PEG400, the content of fatty acids significantly decreased, while the content of phenols, ketones, aldehydes, alcohols, acids had been greatly improved. Especially, the content of phenols increased from 17.65 to 36.33%. When adding Tween80 during HTC, the content of fatty acid increased from 55 to 74.04%. This indicates that Tween80 promoted the hydrolysis of cellulose to produce fatty acid. Meanwhile, the content of alcohols significantly increased from 1.1 to 9.9%. When adding SDBS, the content of aliphatic hydrocarbons significantly increased from 4.1 to 22.59%. Especially, the content of long-chain fatty acids was similar to that in CS/SA. In contrast, the content of acids, aldehydes which were rich in oxygen was almost zero.

Moreover, the absolute content of fatty acid was much higher in the samples of CS/SA/SDBS, CS/SA/TW, CS/SA/SP, reaching 41 mg/g, 59 mg/g and 78 mg/g, respectively. The content of fatty acids and aliphatic hydrocarbons was also much higher in the CS/SA/SDBS and CS/SA/SP than that in the other samples. Moreover, it was clear that the absolute content of phenols, ketones, aldehyde and alcohol was much higher in the sample of CS/SA/SP, indicating that the structure and surface properties were beneficial for the adsorption of all kinds of organic from the hydrolysis of CS. Associated with Fig. 1a, it was clear that SDBS inhibited the hydrolysis of CS, but promoted the conversion of bio-oil into gas and the absorption of solid product for bio-oil in the solution. The main composition of bio-oil was fatty acid and aliphatic hydrocarbons. However, for CS/SA/SP, it significantly enhanced the adsorption of solid product for bio-oil in solution, but inhibited the conversion of bio-oil to gas.

### The fuel properties of corn stover and solid product

#### The pelletization properties of solid product

**Fig. 4:**
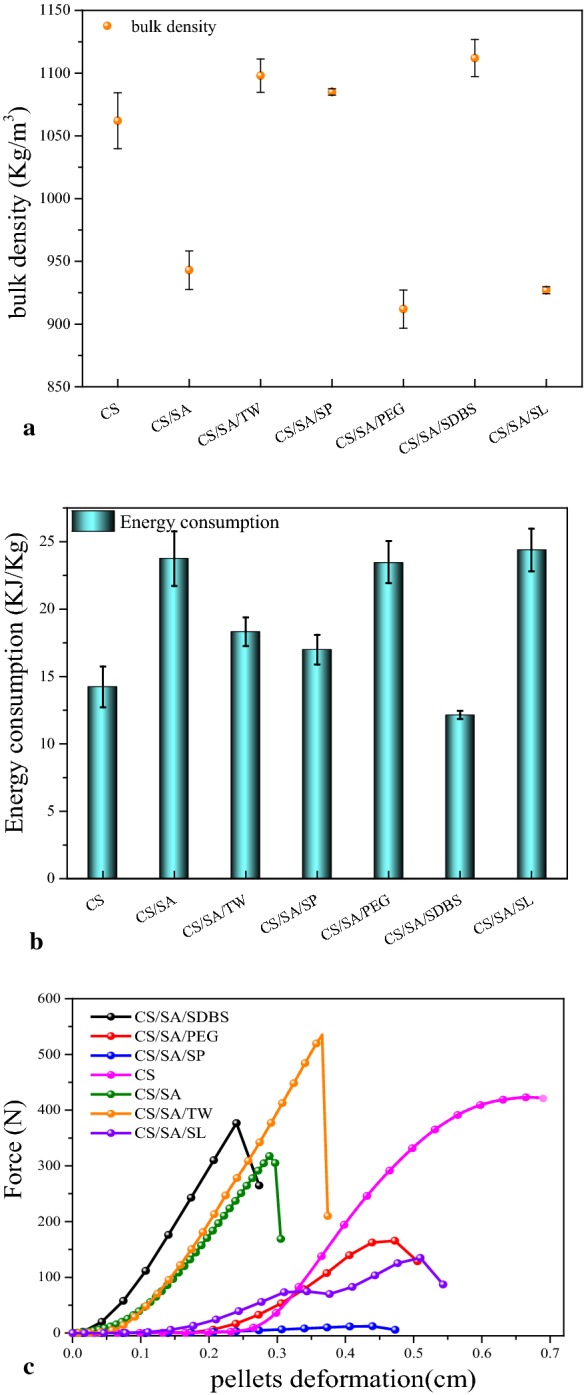
The pelletization properties of solid product and the mechanical strength of pellets. **a** The bulk density. **b** The energy consumption. **c** Radial compressive strength

The pellet density and energy consumption of CS and solid product are shown in Fig. 4a, b. Error bars represented the standard deviations of 3 replicates. The error is caused by the inconsistency nature of CS raw materials and the heterogeneity of CS pellets. It can be seen that the bulk density of the CS directly formed at a temperature of 100 °C under a pressure of 5kN was 1062 kg/m^3^. After HTC treatment, the trend was clearly observed in Fig. 4a. For the controlled sample CS/SA as well as the CS/SA/PEG and CS/SA/SL, the bulk density decreased significantly from 1062 kg/m^3^ to 943 kg/m^3^, 912 kg/m^3^ and 927 kg/m^3^, respectively. Because the removal of chemically bonded water and low-melting point compounds during HTC cause the deficiency of binding agent in solid product [21]. However, the bulk density of solid product from CS/SA/TW, CS/SA/SP and CS/SA/SDBS was 1098 kg/m^3^, 1085 kg/m^3^, and 1112 kg/m^3^, respectively, which was much higher than the one without surfactant (just 943 kg/m^3^ for CS/SA), even higher than CS. Combined the oil content in solid product (in Fig. 1a) and the composition of bio-oil (in Fig. 3), it can be induced that the more the amount of oil in solid product was, the higher the bulk density of the samples was. The addition of SP, TW and SDBS increased the amount of hydroxyl group, low melting and low-softening point materials such as alcohols and fatty acid due to the increase of bio-oil, which exceeded the loss amount of CS raw materials during the HTC.

Moreover, the energy consumption was about 14 kJ/kg for CS. After HTC, the energy consumption during pelleting increased to 23 kJ/kg. It was widely accepted that H– bonding at lignin and hemicellulose surface was the main binding during the pelletization [22]. The decomposition of hemicelluloses and lignin reduced the plasticity of sawdust, which contributed to the increased energy consumption during pelleting [22]. The removal of some small-molecule substances possessed –H bonding in cellulose and hemicellulose caused the increase of energy consumption of pelletization.

The addition of different surfactants during HTC also played a significant role in the properties of solid product pelleting. Compared with the sample of CS/SA, the addition of PEG and SL had a slight increase in energy consumption. However, the addition of Tween80, Span80, SDBS during HTC significantly improved the characteristics of pelleting of solid product. Especially, when adding Tween80, Span80 and SDBS, the energy consumption of solid product during pelleting significantly decreased to 18 kJ/kg, 16 kJ/kg, and 13 kJ/kg, respectively, which was much lower than that of CS/SA without surfactant. It was possible that the addition of surfactants improved the content of bio-oil in solid product. Especially, those bio-oil including acids, alcohols and ketones, were coated on the surfaces of solid product, resulting in an increase of plasticity. Most of the compounds in oil were with lower melting and softening point, which was beneficial for the pelleting properties. Especially, the increase of fatty acids and aliphatic hydrocarbons in oil played the role of promoting the lubrication and adhesive in the pelletization.

At present, most of the research found that most solid product from HTC showed lower bulk density and higher energy consumption than the raw materials [23, 24]. In our research, HTC combined with surfactant significantly increased the bulk density and decreased the energy consumption of pelletization.

The relationship between radial pressure resistance and deformation of pellet samples is shown in Fig. 4c. It can be seen that the plasticity of CS pellet was the strongest during all samples. At first, the deformation occurred in the process of compressive resistance. With the gradual increase of compressive force, the maximum crushing force was 422 N. Meanwhile, the plastic decreased significantly after HTC. Because most of the cellulose and hemicellulose in corn stover was hydrolysis after hydrothermal carbonization which resulting in the brittleness of hydrochar.

For CS/SA sample, when deformation occurred, the force rises linearly until it broke. The maximum crushing force was 305 N. Compared with CS, the maximum compressive force increased significantly after adding SDBS and Tween80 during HTC. Especially, when adding Tween80, the maximum breaking force reached 534 N, exceeding CS. Noticeably, the maximum crushing force of solid product from CS/SA/SP was only 12 N. It was because that the oil in the CS/SA/SP solid product was 45.49% in Fig. 1a. The main components of oils were fatty acids, reaching 78 mg/g CS. The pellet was soaked in waxy fatty acids due to the high content of oil. The conclusion was confirmed by the images of SEM. Therefore, the strength of pellet was similar to the wax, which was much weaker than CS. However, for TW and SDBS, the oil content (especially the fatty acids) was not too high to decrease the strength significantly. On the contrary, for the samples of CS/SA/PEG and CS/SA/SL, the fatty acid content was lower than other sample, resulting in low bulk density and strength. This indicated that fatty acid in the suitable range of 41–78 mg/g CS was beneficial for improving the strength and plasticity of pellet.

In summary, oil in solid product obtained via adding surfactant gave a high heat value, bulk density, strength and plasticity, and low energy consumption for pelleting of solid product.

#### Grindability

**Fig. 5:**
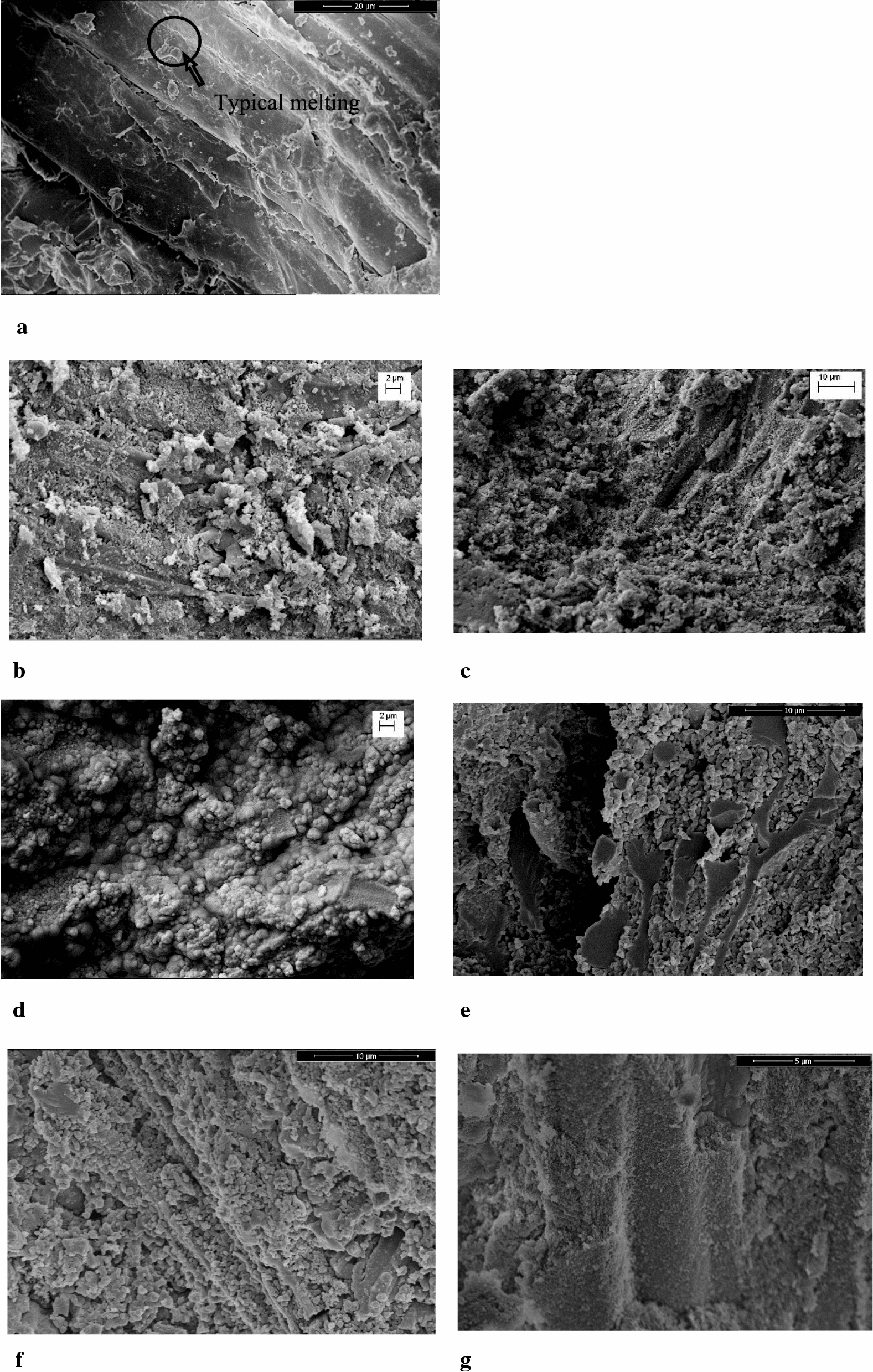
SEM image of pellets obtained with different surfactants (**a** material; **b** SDBS; **c** PEG400; **d** Span80; **e** Tween80; **f** 2% sulfuric acid; **g** SL)

The forming mechanism of CS pellets as well as solid product has a significant effect on the bulk density and strength of pellets. The bonding process of solid product particle obtained via HTC with different surfactant can also be illustrated by SEM in the Fig. 5. It was widely accepted that a solid bridge, thermal fusion, the materials with low melting point and rich H– as binder were contributed to binding mechanism of biomass. The surface was smooth and there was no obvious cracking between the particles due a solid bridge, thermal fusion, and the bonding of the materials in CS [25, 26]. The results were consistent with Kaliyan [26] who found that the fiber of corn stover was closely connected due to the high pressure during pelleting, and the natural compounds in corn stover were squeezed out of biomass cells and formed a solid bridge among corn stover particles. These solid bridges improved the strength of pellets. However, for the solid product, the materials with low melting point and rich H– as binder were decomposed during HTC, resulting in the disappearance of the binder. The surface of CS/SA was roughness and interparticle distance was far. Furthermore, for the CS/SA/SL and CS/SA/PEG pellets, the content of the fatty acids with low melting as binder was very low, and the binding of particles depended on the thermal fusion. Therefore, the particles were intricately piled up.

Moreover, for CS/SA/SP, the oil content was very high that the solid product particles were soaked in the oil. Although the oil was beneficial for bonding the particles, it decreased the strength of pellets due to lacking the main skeleton composed of solid product. Especially, for CS/SA/SDBS and CS/SA/TW pellets, the solid bridge was obvious. It was that an adsorption layer was produced by the oil in the particles, which attached the particles by effectively reducing roughness and interparticle distance. Moreover, the content of oil in solid product was high enough to replace the role of compounds removed during HTC and formed solid bridge among particles. Those made the pellets to possess similar strength and density like CS pellets. Moreover, for CS/SA/TW pellet, there was dense skeleton surrounding the particles which enhanced the strength of pellets. Moreover, Bika et al. [27] explained the forming mechanism of a solid bridge. The binding of particles was from the bonding force at interface between the solid particles and the binder, and the cohesive force in the binder. The existence of liquids such as free water between particles causes cohesive force between particles. If the dispersity was high in the liquid binder, the liquid binder would spread in the gap between the particles and form a liquid bridge through the role of capillary action and viscous force. After drying, the liquid evaporated from the bridge and left a solid bridge between the particles. Therefore, the surface properties played an important role in the pelleting. The surface of solid product was different due to the difference of content and composition of oil coating the solid product, which played a key role in the pelleting of solid product.

#### Hydrophobicity

**Fig. 6:**
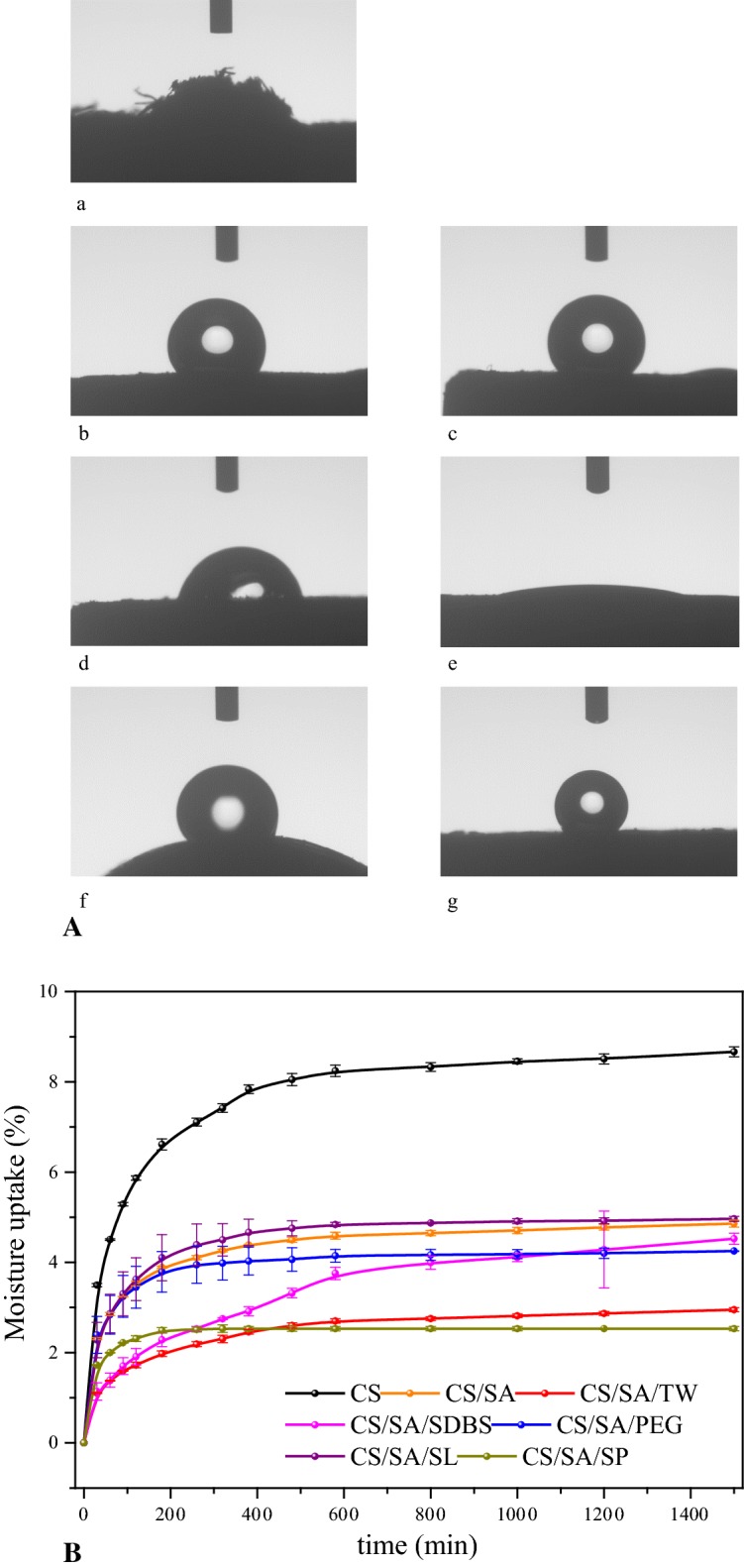
The properties contact angle and water sorption of CS and solid product. **A** The Contact angle of CS and solid product. **B** Water sorption by pellets over 25 h at an air humidity of 70% and 30 °C

The contact angle indicated the strength and hydrophobicity of the pellets. The contact angle of solid product as well as CS is shown in Fig. 6A. The hydrophilic properties of the CS pellets were very strong. Therefore, the state of CS pellet could not be maintained after absorbing the water. In contrast, the pellet changed into hydrophobicity after HTC and the contact angle of the CS/SA sample was 138.2°. It was because that the OH– in the hemicellulose and cellulose, which was the key point for the hydrophilicity in CS raw materials, was removed during HTC. Moreover, after the addition of various surfactants, the contact angle of the solid product pellets changed significantly. Especially, the contact angles of CS/SA/TW, CS/SA/SP, CS/SA/SL, CS/SA/SDBS and CS/SA/PEG were 116.7°, 127.4°, 99.7°, 22.3°, and 133.8°, respectively. It was obvious that the hydrophobicity varied with the surfactants, indicating that adding surfactant can effectively change the surface properties of solid product. The content and composition of bio-oil adsorbed on the surface of solid product were different when adding different surfactant. Especially, the addition of Tween80, Span80, PEG400, solid product pellets still retained hydrophobic properties. However, with the addition of SL and SDBS, the contact angle greatly reduced. The surface of solid product changed from hydrophobicity to hydrophilicity. The SDBS was hydrophilic surfactants, which adhered to the surface of solid product and changed the surface properties of pellet. Tween80 was also a strong hydrophilic surfactant, but the content of oil absorbed on the solid product was much lower than SDBS (in Fig. 1a), indicating that no or very less Tween80 adhered to the surface of solid product. Therefore, the change of hydrophobicity properties was not obvious.

In summary, the hydrophilicity of CS can be significantly turned to hydrophobicity via HTC. However, adding different surfactant during HTC can change surface properties of solid product to hydrophobicity or hydrophobicity, depending on the HBL vale of surfactants.

#### Water sorption

Figure 6B showed the water sorption of treated samples as well as CS. For CS raw material, when reaching the water absorption balance, the water adsorption capacity was about 9%. It was accepted that the adsorption of water increases due to the formation of large voids after cell wall decomposition during HTC. In contrast, the equilibrium water absorption of the pellets of solid product was in the range of 2.5–4.96%, which was much lower than CS raw materials. The results were consistent with the research of Gray [28] and Järvinen [29] who found that torrefied char or solid product was more hydrophobic than the original biomass feedstock. It means that part of hydroxyl groups was removed during HTC [30]. Moreover, the squeezing of solid product in die channels during pelletization also would reduce the size of these voids and reduce the pellets’ water absorption [31]. The water adsorption was in the following order: CS/SA/SP < CS/SA/TW < CS/SA/SDBS < CS/SA/PEG < CS/SA < CS/SA/SL. This was consistent with the results of contact angle in Fig. 6A. It was possible that the pellet particles were coated by oil, which is a disadvantage for the adsorption of water. Especially, the adsorption water content varied with the content and composition of oil coated in the particles. However, the correlation was not clear now. Especially, the contact angle of CS/SA/SDBS sample was less than 90° and exhibited hydrophilic properties. But the adsorbed water amount was only 4.52%, which was still much lower than that of CS raw material possessing the same hydrophilicity. It was well known that the SDBS was hydrophilic surfactants, which adhered to the surface of solid product and changed the surface of solid product from hydrophobicity to hydrophilicity. However, some of the voids in solid product were filled with oil and the removal of H– bond prevented the uptake of water. In summary, the uptake of water was much low for HTC-treated samples, which was advantageous for storage and transportation.

#### The combustion properties and kinetic analysis of pellets

**Fig. 7:**
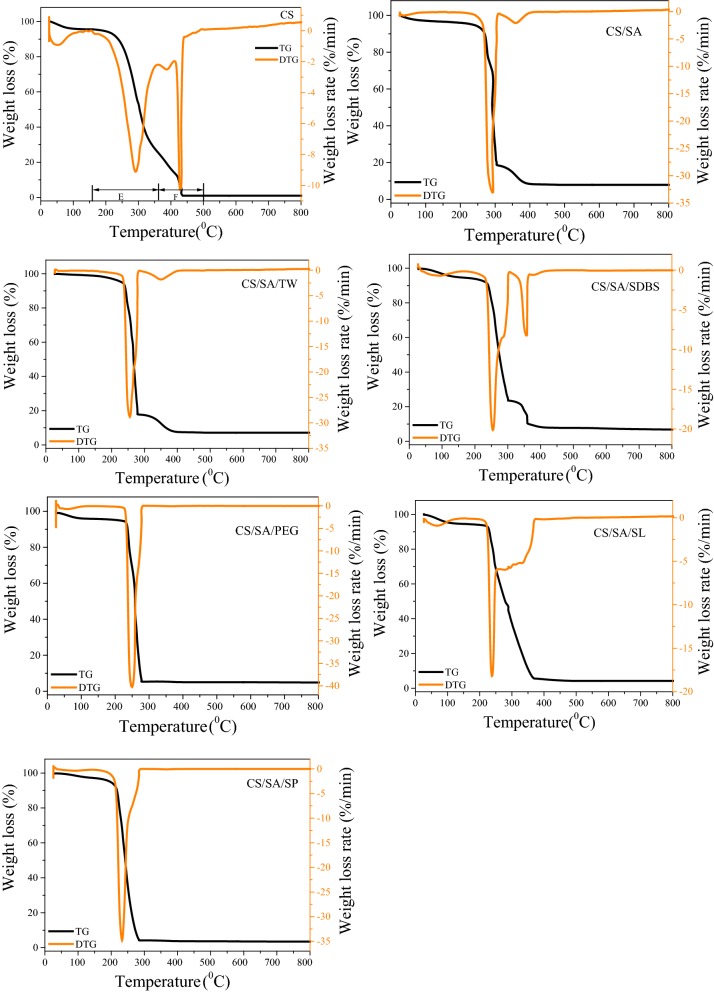
TG curve of CS and solid product obtained from hydrothermal carbonization in different condition

Figure 7 and Table 3 show the combustion characteristics of solid product as well as CS. Adding different surfactant during HTC, the combustion-related characteristics of solid product had undergone significant changes. Specifically, there were two stages for the main combustion of CS. They focused on 156–354 °C and 354–503 °C, respectively, which corresponded to the release and combustion of the volatile matter (1st stage) and the combustion of fixed carbon (2nd stage), respectively [32–34]. For all solid product obtained via HTC combined with surfactant, the combustion temperature in the second stage decreased. Moreover, for CS/SA/SP and CS/SA/PEG samples, the 2nd combustion peaks disappeared. However, the content of fixed carbon CS/SA/SP and CS/SA/PEG was 26.64% and 40.58%, respectively. Moreover, the maximum combustion rate was 40%/min and 35%/min, which was higher than other samples. The combustion temperature for VM and FC in CS/SA/SP and CS/SA/PEG was similar, focusing on 200–300 °C. The temperature was lower than CS and CS/SA sample. The results were consistent with maximum combustion rate Mr and ignition temperature Ti in Table 3. Mr of CS/SA/SP and CS/SA/PEG was fast than other samples and Ti was lower than other sample. The conversion of two individual peaks to one sharp peak with a significant shift to low temperature zone would be a great advantage for fast and easy heating and stability [35].

**Table 3:** Combustion characteristic parameters of samples

Sample	SN (10^−11^ K^−3^ min^−2^)	Ar (%·min^−1^)	Mr (%·min^−1^)	Ti (K)	Th (K)	τ0 (min)	Ci/10^−4^(K^−2^ min^−1^)	Cr/10^−7^(K^−2^ min^−1^)
CS	2.13	4.62	9.12	525.35	716.15	40.6	1.3124	3.30
CS/SA	11.30	6.89	33.10	554.45	656.85	35.7	3.2381	10.77
CS/SA/TW	10.10	6.09	28.81	516.05	651.65	35.1	3.1369	10.82
CS/SA/SDBS	7.97	6.72	20.15	511.85	648.25	33.4	2.1105	7.69
CS/SA/PEG	100.71	36.76	40.28	489.65	613.15	23.2	4.2637	16.80
CS/SA/SL	7.42	6.39	18.24	499.05	629.95	33.1	1.8859	7.32
CS/SA/SP	28.98	11.28	34.78	490.65	562.15	26.6	4.6898	14.45

*SN* integrated combustion characteristics index, *Ar* average reaction rate (d*w*/d*t*)_mean_, *Mr* Maximum reaction rate (d*w*/d*t*)_max,_ *Ti* ignition temperature, *Th* burnout temperature, *τ0* burnout time, *Ci* index of ignition characteristics, *Cr* index of combustible characteristics

Moreover, SN, Ar, Mr, Ti, and Th were critical factors to evaluate the combustion of solid product. The combustion characteristic index Cr, the ignition characteristic index Ci, and the comprehensive combustion characteristic index SN of CS were 3.30 × 10^−7^ (K^−2^ min^−1^), 1.3124 (K^−2^ min^−1^), and 2.13 × 10^−11^(K^−3^ min^−2^), respectively. After HTC, the combustion index Cr of CS/SA reached 10.77 × 10^−7^ (K^−2^ min^−1^). The comprehensive combustion characteristic parameter increased to 11.30 × 10-11 (K^−3^ min^−2^), which was nearly 5 times as much as CS. The decomposition of cellulose and hemicellulose during HTC was the main reason. Moreover, when adding surfactant, the Ti and Th of solid product were significantly lower than that of CS/SA and CS. It was possible that surfactant promoted the decomposition of CS and enhanced the specific surface area and total pore volume of solid product, which was beneficial for the combustion of solid product [15]. Moreover, the absorbed oil on the solid product played the role of combustion supporting agent, which would also promote the combustion. It was accepted that the lower Ti and Th were, the easier the combustion was. Those induced that HTC combined with surfactant was not only beneficial for improving the heat value but also enhancing the combustion properties.

Especially, when adding PEG, the combustion index Cr of 16.80 × 10^−7^ (K^−2^ min ^−1^) was 5 times as much as CS. Noticeable, The integrated combustion characteristic SN increased from initial 2.13 × 10 ^−11^ (K^−3^ min^−2^) to 100.71 × 10^−11^ (K^−3^ min ^−2^), increasing by nearly 50 times, which was much higher than that in other research in the field of biomass fuel. Wang et al. [31] found that the comprehensive combustion characteristic index of wheat straw as a pellet fuel was only 3.9 × 10^−11^ (K^−3^ min^−2^). He [32] studied the transformation of sewage sludge into a clean solid by HTC. The comprehensive combustion characteristics index of sewage sludge fuel was 0.8173 × 10^−11^ (K^−3^ min^−2^). Those induced PEG400 significantly improved the combustion properties of CS, especially, the average burning rate and maximum burning rate of CS. Meanwhile, the ignition point of CS/SA/PEG was 489.65 K, which was lower than that of other samples. The burn-out temperature was 613.15 K. The combustion centralized in the range of 228–276 °C with the maximum mass loss rate 40.28% min^−1^ and mass loss 84%. The combustion properties of CS/SA/PEG were similar with diesel oil, inducing that solid product was a promising fuel with high heat value and good combustion properties.

Moreover, integrated combustion characteristics index SN of solid product produced with different surfactant were in the order: CS/SA/PEG > CS/SA/SP > CS/SA > CS/SA/TW > CS/SA/SDBS > CS/SA/SL. Compared with CS/SA, after adding surfactant, the burning time, ignition and burning out temperature were reduced. It indicated that the surfactant promoted the formation of oil in solid product which was beneficial for igniting and promoting the combustion. They varied with the composition of oils as well as the chemical properties of solid product.

For CS/SA sample, the activation energy of solid product showed the increase due to the decomposition of cellulose and hemicellulose during HTC. Moreover, the release of volatile matter was also one of the reasons for the activation energy increase. The result was also consistent with that the volatile content of CS, which slightly decreased after HTC. It was also well known that the activation energy could be mainly enhanced via improving the surface area and porosity of solid product [10]. Moreover, the activation energy of CS/SA/SP significantly increased from 97.91 to 162.77 kJ/mol. It was because that the oil absorbed on the surface of solid product blocked the pore, which decreased the surface area and porosity of solid product. Besides, the content of phenols, which was attributed to the increase of activation energy, was much higher in the sample of CS/SA/SP. Furthermore, compared with CS/SA, the activation energy decreased for the CS/SA/TW, CS/SA/SDBS, CS/SA/PEG, CS/SA/SL. There were three reasons to explain the phenomenon: (1) surfactant promoted the decomposition of CS and formed porosity on solid product. (2) The bio-oil absorbed on the surface played an important role in the decrease of activation energy. Especially, some compounds in the bio-oil act as the combustion supporting agent, which significantly decreased the activation energy. (3) Decomposition of residual small molecular substances in hydrothermal carbon was also one of the reasons. Moreover, the low active energy for solid product showed that surfactant combined with HTC improved the combustion properties.

### The mechanism of improvement of pelletization and combustion behavior of corn stover via hydrothermal carbonization combined with surfactant

**Fig. 8:**
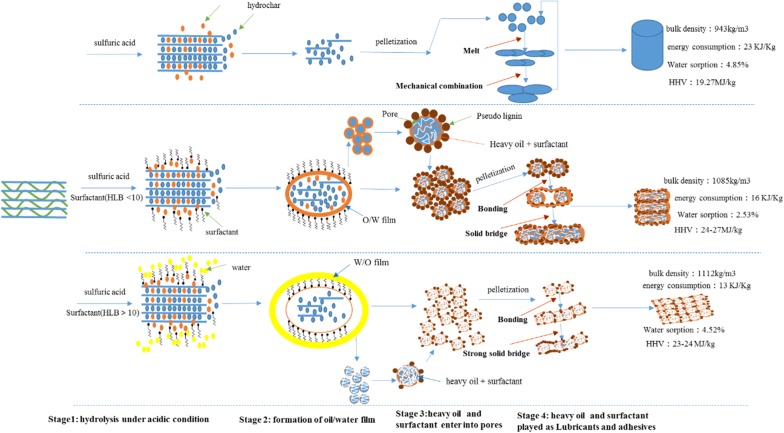
The mechanism of improvement of pelletization and combustion behavior of corn stover via hydrothermal carbonization combined with surfactant

The mechanism of improvement of pelletization and combustion behavior of CS via HTC combined with surfactant is shown in Fig. 8. The HTC of CS was under acidic condition. All hemicellulose and part of lignin and cellulose were decomposed to produce bio-oil and water during HTC. Moreover, the surfactant (HLB < 10) connected with bio-oil via oleophilic groups, gradually formed an O/W film on the surface of CS particles. In contrast, when adding surfactant (HLB > 10), the W/O film was formed on the surface of solid product particles [36]. The film became thicker and thicker due to the accumulation of bio-oil. Meanwhile, the pseudo-lignin was formed due to the polycondensation of organic matter from hydrolysis of hemicellulose and cellulose [37]. Furthermore, the pore in the solid product was formed due to the decomposition and removal of hemicellulose and cellulose. The bio-oil and surfactants in the film gradually entered into the pores of solid product under the role of in situ pressure at the end of HTC. Meanwhile, the carbon microsphere of pseudo lignin was absorbed on the surface of solid product, which was beneficial for improving the heat value of solid product due to the high C content. The bio-oil and surfactant on the surface or in the pores of solid product significantly improved the pelletization of solid product, such as the decrease of the energy consumption and the enhancement of the bulk density. It was possible that the bio-oil and surfactant played as lubricants and adhesive to enhance the role of solid bridge among particles and the bonding of functional groups such as OH– and H–. Especially, the W/O film was more beneficial for forming strong solid bridge, which was in favor of decreasing the energy consumption during pelletization and increasing the bulk density of pellets. Moreover, the composition with low ignition point in the bio-oil which was absorbed in the pellets played the role of ignition agent, which decreased the ignition point of pellets. Meanwhile, the absorbed bio-oil and pseudo-lignin with high C content significantly increased the HHV of pellets. All of these were beneficial for the improvement of pellets combustion and pelletization properties.

## Discussion

Large-scale application of biomass as solid fuels for gasification and renewable products was restricted due to low bulk density, energy density as well as high hydrophilicity and cost of transport, handling and storage. Therefore, pelletization combined with torrefaction was widely investigated to overcome the drawback and save from cost in commercial application of biomass. However, it needs higher mold temperature and mechanical energy to compress torrefied TS into pellets with the same hardness and density as regular pellets, which significantly improves the cost of pelletization.

1. The mold temperature of pelletization for torrefied biomass was higher than untreated biomass, even in the range of 125–180 °C [38]. Meanwhile, the mechanical energy consumption for compressing torrefied biomass into pellets was much higher than the common biomass with the similar hardness and density [39]. Therefore, in our articles, the surfactant was mixed with the biomass during the wet torrefaction, which made the mold temperature decreased to 100 °C and the mechanical energy consumption decreased from 23 to 13 kJ/kg. Moreover, the bulk density increased from 943 to 1112 kg/m^3^. Those significantly improved the quality of pellets and decreased the cost of torrefied biomass pelletization.
2. Moreover, it was widely accepted that the HHV of biomass pellet (14–19 MJ/kg) was much lower than fossil fuel (41.87 MJ/kg) or coal (29 MJ/kg). In our articles, the HHV of torrefied pellets increased from 19.27 to 27 MJ/kg. Meanwhile, the combustion properties of CS pellet such as ignition and burning temperature as well as kinetic parameters were significantly improved after HTC treatment combined with surfactant. The combustion properties of CS/SA/PEG were similar to diesel oil.
3. It was widely accepted that a solid bridge, thermal fusion, some materials with low melting point and rich H– as binder were contributed to pelletization mechanism for biomass [25, 26]. However, the pelletization mechanism of CS treated with HTC combined with surfactant were not exactly the same. The results show: (1) surfactant promotes the formation of pseudo-lignin and the absorption for oil with low O and high C during HTC in acidic condition, which improves the energy density of solid product. (2) The oil in solid product plays the role of lubricant and binder, which reduces the mold temperature, energy consumption and improves bulk density and pellets strength of pellets. (3) Surfactant combined with HTC improved the combustion characteristic of solid product due to the role of absorbed bio-oil as igniting agent and the improvement of surface and porosity.

## Conclusion

HTC combined with surfactant was employed to overcome the drawbacks of low bulk density, high cost of transport and storage of CS and improve the pelletization and combustion properties of solid product. The results show that the surfactant promotes pseudo-lignin formation and absorption for oil with low O and high C during HTC, which improves the energy density of solid product. Furthermore, the oil in solid product plays the role of lubricant and binder, which reduces the negative effect of high energy consumption, low bulk density and weak pellets strength caused by HTC during pelletization. Moreover, surfactant combined with HTC improved the combustion characteristic of solid product such as ignition and burning temperature as well as kinetic parameters due to the bio-oil absorbed and the improvement of surface and porosity. Furthermore, the large-scale experiments in a real market as well as reactor design will be investigated in the future.

## Material and method

### Material preparation

The particle size of the corn stover (CS) materials was 0.25–0.35 mm, which was purchased from Yangjiang city of Guangdong Province, China. Before the reaction, it was dried at 70 °C for 24 h. H_2_SO_4_ (98%), Tween80 (TW, HBL = 15), Span80 (SP, HBL = 4.3), sodium dodecylbenzenesulfonate (SDBS, HBL = 10.6), sodium lignosulfonate (SL, HBL = 8.9), PEG400 (HBL = 11.6) were purchased from Fuchen chemistry Limited Liability Company. H_2_SO_4_ (98%) was added into deionized water to maintain the concentration of acid (2%) and surfactant (2%) based on the mass of de-ionized water.

### Experimental procedure

#### Hydrothermal experiment

The hydrothermal carbonization was prepared in a 500-ml batch autoclave at 210 °C for 60 min with a heating rate of approximately 4.3 °C/min, which was similar to the previous research [5]. About 20-g CS, 6-g 98% sulfuric acid, 6-g surfactant (Tween 80, Span80, sodium dodecylbenzenesulfonate, sodium lignosulfonate, PEG 400) and 300-ml deionized water were put into the autoclave. The ratio of solid to liquid was 1:15 and the stirring speed was 600 rpm. High-purity nitrogen was used to remove air from the autoclave. When the reaction finished, the autoclave was placed in air and cool to 85 °C. Then put 2% NaOH solution into the mixture of solid products till the pH was close to 7. Then, the solid–liquid mixture was separated via filter. The solid product was dried at 105 °C for 24 h and labeled as CS/SA (when using Tween80, Span80, sodium dodecylbenzenesulfonate, sodium lignosulfonate, PEG400, the solid products were labeled as CS/SA/TW 80, CS/SA/SP80, CS/SA/SDBS, CS/SA/SL, CS/SA/PEG, respectively). Moreover, to analyze the content and composition of bio-oil in the CS/SA, 1-g solid product was immersed in dichloromethane at room temperature and stirred for 24 h. The liquid product was analyzed by GC–MS. In this study, the bio-oil was the filtered bio-oil. The “oil in solid product” was the oil extracted from solid products via DCM.

#### The process of pelletization and combustion was similar in our previous research [5]

The specific process was briefly introduced as follows:

*Pelletization* The compression test of biomass particles uses an electronic universal testing machine. Approximately 1 g of the sample was added in a mold having a diameter of 10 mm and a length of 70 mm. When the mold was heated to 100 °C, the sample was compressed at a pressure of 5 kN and retained for 5 s. Then, the pellet was taken out and collected in a sealed bag. Repeat 5 times for each sample to ensure the accuracy of the experiment.

$$ {\text{Energy}}\;{\text{consumption }} = \, {W \mathord{\left/ {\vphantom {W m}} \right. \kern-0pt} m} \, = \frac{{\int {f{\text{d}}s} }}{m} $$

where *W*, *m*, *s*, *f* were the specific energy consumption of the compression process (kJ/kg), total energy consumption (J), pellet mass (g), position move (mm), and pressure (kN), respectively.

*Combustion* Combustion analysis was carried on the TG-DSC 449c thermogravimeter. About 10 mg of the raw material and solid product were added into a crucible of Al_2_O_3_. Air was used as a purge gas with a flow rate of 80 ml/min. The heating procedure was that the temperature increased from 26 to 900 °C at a heating rate of 10 °C/min.

### Product analysis for physical and chemical properties of solid product and CS was similar in our previous research [11]

The physical and chemical properties of the samples, such as moisture content; ash content; volatile matter, fixed carbon, using automatic industrial analyzer (Changsha Youxin Instrument Manufacturing Co., Ltd.) with reference to GB/T28731-2012.

Calorific value was tested using an YX-ZR Skyhawk Automatic Calorimeter (Changsha Youxin Instrument Manufacturing Co., Ltd.).

The composition of bio-oil was tested on an Agilent 7890B-5977A GC–MS (Gas Chromatography–Mass Spectrometer) system at the Instrumental Analysis & Research Center South China Agricultural University (Guangzhou, China). A column of Ptx-Wax (30.00 m × 0.25 mm × 0.25 μm) was equipped on GC–MS. The content of the main component contained in the liquid product was calculated as the ratio of the peak area of the identification component to the peak area of the total component minus the peak area of the solvent dichloromethane. [5, 11].

The contact angle (CA) was tested in DSA100 (Germany) [40]. The pellets were placed on the sample stage, and the flat surface of the pellets was subjected to a contact angle test, and the height of the sample stage and the focal length of the lens were adjusted to an appropriate position. When the droplets were completely dropped on the pellets, the recording was taken after 30 s. The contact angle of the droplets with the pellets at this time was the contact angle measured in this study.

### The calculation of kinetics of solid product combustion was according to the previous research [5]

Generally, kinetic equation for solid combustion was as following:

$$ {\text{d}}\alpha /{\text{d}}t \, = \, kF\left( \alpha \right) $$

*k* = *A*exp(− *E*/*RT*) according to Arrhenius’ law.

Moreover, d*α*/d*t* = *A*exp(− *E*/*RT*) *F* (*α*), *β* = d*T*/d*t*, *β* was the heating rate.

Therefore, d*α*/d*t* = *A*/*β* * exp(− *E*/*RT*) * *F* (*α*) was used as the kinetic analysis equation.

For a better understanding of the enhancement of surfactant combined with HTC on combustion behavior of CS and solid product, the kinetic parameters were investigated via the data of TGA. As usual, the *n*th-order kinetics model was used to evaluate the combustion of solid fuel [32]. In this study, kinetics model Fn = (1 − *α*)^*n*^, Cn − *X* = (1 − *α*)^*n*^(1 + *k*cat*X*) in the software of NETZSCH Thermodynamics Analysis was used to fit the combustion kinetic of solid product and CS. The pre-exponential factor (*A*), activation energy (*E*), *n*th-order of reaction (*n*) and correlation coefficients (*R*) are shown in Table 4. For all samples, high correlation coefficients (*R*^2^ > 0.99) induced that the *n*th-order kinetic mode (Fn, Cn) was in good fit with the TGA data. Thus, the *n*th-order kinetic mode (Fn, Cn) was suitable for describing the combustion process of CS and solid product.

**Table 4:** Kinetic parameters for CS and solid product

Sample	*E* (kJ/mol)	Log *A* (s^−1^)	*n*	*R* ^2^
CS	77.75	4.8595	1.4	0.996
CS/SA	97.91	5.8455	2.3	0.997
CS/SA/TW	72.72	3.2487	2.5	0.999
CS/SA/SDBS	56.14	2.3073	1.4	0.998
CS/SA/PEG	60.27	2.3433	2.1	0.999
CS/SA/SL	51.41	2.3349	1.2	0.997
CS/SA/SP	162.77	14.7603	2.2	0.999

### Calculation formula

The calculation formula of energy parameter is shown in Table 5.

**Table 5:** The calculation formula of energy parameter

Term	Equation
Solid recovery (%)	$\frac{{{\text{Mass}}\;{\text{of}}\;{\text{dried}}\;{\text{solid}}\;{\text{product}}}}{{{\text{Mass}}\;{\text{of}}\;{\text{dried}}\;{\text{initial CS}}}}\;*\;100$
Carbon recovery efficiency (%)	$\frac{{{\text{carbon }}\;\left( {\text{\% }} \right)\;{\text{in}}\;{\text{solid}}\;{\text{product}}\;*\;{\text{solid}}\;{\text{product}}\;{\text{weight}}}}{{{\text{carbon}}\;\left( {\text{\% }} \right)\;{\text{in}}\;{\text{CS}}\;*\;{\text{CS}}\;{\text{weight}}}}\;*\;100$
Fixed carbon recovery efficiency (%)	$\frac{{{\text{Fixed}}\;{\text{carbon}}\;\left( {\text{\% }} \right) \;{\text{in}}\;{\text{solid product}}\;*\;{\text{solid}}\;{\text{product}}\;{\text{weight}}}}{{{\text{Fixed}}\;{\text{carbon}}\;\left( \% \right) \;{\text{in}}\;{\text{CS}}\;*\;{\text{CS}}\;{\text{weight}}}}\;*\;100$
Energy densification efficiency (%)	$\frac{{{\text{calorific}}\;{\text{value}}\;{\text{of}}\;{\text{solid}}\;{\text{product}}}}{{{\text{calorific}}\;{\text{value}}\;{\text{of}}\;{\text{CS}}}}*100$
Energetic retention efficiency (%)	$\frac{{{\text{calorific}}\;{\text{value}}\;{\text{of}}\;{\text{solid}}\;{\text{product}}}}{{{\text{calorific}}\;{\text{value}}\;{\text{of}}\;{\text{CS}}}}\;*\;{\text{Solid}}\;{\text{recovery}}$
HHV improvement (%)	$\frac{{{\text{HHV}}\;{\text{of}}\;{\text{solid}}\;{\text{product}} - {\text{HHV}}\;{\text{of}}\;{\text{CS}}}}{{{\text{HHV}}\;{\text{of}}\;{\text{CS}}}}\;\;*\;\;100$

## Supplementary information

**Additional file 1: Fig. S1.** FT-TR analysis of CS and solid productions. **Table S1.** Band assignments in the infrared region of lignin. **Table S2.** the composition of oil eluted from solid product (relative content %). **Table S3.** the composition of oil eluted from solid product (absolute content mg/g CS).

## Data Availability

The datasets used and analyzed during the current study were available from the corresponding author on reasonable request.

## Abbreviations

HTC: hydrothermal carbonization
CS: corn stalk
SDBS: sodium dodecylbenzenesulfonate
PEG: PEG 400
SL: sodium lignosulfonate
TW: Tween80
SP: Span80
CS/HT: hydrothermal carbonization of corn stalk
CS/SA/TW: hydrothermal carbonization of corn stalk under 2% sulfuric acid and 2% Tween80
CS/SA/SP: hydrothermal carbonization of corn stalk under 2% sulfuric acid and 2% Span80
CS/SA/SDBS: hydrothermal carbonization of corn stalk under 2% sulfuric acid and 2% sodium dodecylbenzenesulfonate
CS/SA/PEG: hydrothermal carbonization of corn stalk under 2% sulfuric acid and 2% PEG400
CS/SA/SL: hydrothermal carbonization of corn stalk under 2% sulfuric acid and 2% sodium lignosulfonate
HHV: heat high value
ERE: energetic retention efficiency
CRE: carbon recovery efficiency
HLB: hydrophile lipophilic balance
*E*: activation energy
*A*: preexponential factor
*n*: order of reaction
*R*: correlation coefficient
CA: contact angle
SN: integrated combustion characteristics index
Ar: average reaction rate (d*w*/d*t*)_mean_
Mr: maximum reaction rate (d*w*/d*t*)_max_
Ti: ignition temperature
Th: burnout temperature
τ0: burnout time
Ci: index of ignition characteristics
Cr: index of combustible characteristics
