# Supplementary material for: Improvement of corn stover fuel properties via hydrothermal carbonization combined with surfactant
Source: Biotechnol Biofuels. 2019 Oct 17;12:249. doi: 10.1186/s13068-019-1581-x (PMC6796392; doi:10.1186/s13068-019-1581-x)
Supplement: Supplementary file 1 — Additional file 1: Fig. S1. FT-TR analysis of CS and solid productions. Table S1. Band assignments in the infrared region of lignin. Table S2. the composition of oil eluted from solid product (relative content %). Table S3. the composition of oil eluted from solid product (absolute content mg/g CS). [file 13068_2019_1581_MOESM1_ESM.docx]

Fig. S1 FT-TR analysis of CS and solid productions

Table S1 Band assignments in the infrared region of lignin.

| Wavenumber/cm^−1^ | Band assignment |
| --- | --- |
| 3460—3412 | O-H stretching |
| 3000—2842 | C-H stretch in methyl and methylene group |
| 1738—1709 | C=O stretch in unconjugated ketone, carbonyl and ester groups |
| 1605—1593 | Aromatic skeleton vibrations plus C=O stretching |
| 1515—1505 | aromatic skeleton vibrations |
| 1470—1460 | C-H deformations (asym in CH_3_ and CH_2_) |
| 1430—1422 | aromatic skeleton vibrations combined with C-H in plane deformations |
| 1370—1365 | aliphatic C-H stretching in CH_3_ and phen. OH |
| 1330—1325 | condensed S and G ring |
| 1233—1214 | C-C+C-O+C=O stretching |
| 1110 | aromatic C-H deformation of S units |
| 1035—1030 | aromatic C-H in-plane deformation (G>S) plus C-O deform.  in primary alcohols plus C-H stretching (unconjugated) |

Table S2 the composition of oil eluted from solid product (relative content %)

| Sample | CS/SA | CS/SA/PEG | CS/SA/TW | CS/SA/SDBS | CS/SA/SP | CS/SA/SL |
| --- | --- | --- | --- | --- | --- | --- |
| Fatty acid | 54.49 | 1.14 | 74.04 | 55.00 | 31.18 | 22 |
| Phenols | 17.65 | 36.33 | 6.14 | 4.25 | 20.62 | 32 |
| Ketones | 13.35 | 25.24 | 5.67 | 7.35 | 13.09 | 12 |
| Others | 5.22 | 10.46 | 3.86 | 9.49 | 13.77 | 11.8 |
| Aldehydes | 3.65 | 10.28 | 0.31 | 0.58 | 5.78 | 5.6 |
| Alcohols | 1.10 | 4.90 | 9.22 | 0.33 | 6.18 | 4.8 |
| Acids | 1.54 | 10.73 | 0.13 | 0.74 | 2.35 | 1.8 |
| Aliphatic hydrocarbons | 4.10 | 0.92 | 0.63 | 22.59 | 7.03 | 10 |

Table S3 the composition of oil eluted from solid product (absolute content mg/g CS)

| Sample | CS/SA | CS/SA/PEG | CS/SA/TW | CS/SA/SDBS | CS/SA/SP | CS/SA/SL |
| --- | --- | --- | --- | --- | --- | --- |
| Fatty acid | 26.31 | 0.35 | 40.88 | 58.75 | 77.30 | 11 |
| Phenols | 8.52 | 11.02 | 3.39 | 4.54 | 51.12 | 16 |
| Ketones | 6.45 | 7.65 | 3.13 | 7.85 | 32.45 | 6 |
| Others | 2.52 | 3.17 | 2.13 | 10.14 | 34.14 | 5.9 |
| Aldehydes | 1.76 | 3.12 | 0.17 | 0.62 | 14.33 | 2.8 |
| Alcohols | 0.53 | 1.48 | 5.09 | 0.35 | 15.32 | 2.4 |
| Acids | 0.74 | 3.25 | 0.07 | 0.79 | 5.83 | 0.9 |
| Aliphatic hydrocarbons | 1.98 | 0.28 | 0.35 | 24.13 | 17.43 | 5 |
